# Investigating clinical handover and healthcare communication for outpatients with chronic disease in India: A mixed-methods study

**DOI:** 10.1371/journal.pone.0207511

**Published:** 2018-12-05

**Authors:** Claire Humphries, Suganthi Jaganathan, Jeemon Panniyammakal, Sanjeev Singh, Shifalika Goenka, Prabhakaran Dorairaj, Paramjit Gill, Sheila Greenfield, Richard Lilford, Semira Manaseki-Holland

**Affiliations:** 1 Institute of Applied Health Research, University of Birmingham, Birmingham, West Midlands, United Kingdom; 2 Public Health Foundation of India, New Delhi, Delhi, India; 3 Centre for Chronic Disease Control, New Delhi, Delhi, India; 4 Sree Chitra Tirunal Institute of Medical Sciences and Technology, Trivandrum, Kerala, India; 5 Hospital Administration, Amrita Institute of Medical Sciences, Kochi, Kerala, India; 6 Academic Unit of Primary Care, University of Warwick, Coventry, UK; 7 Centre for Applied Health Research and Delivery, University of Warwick, Coventry, UK; Nord University, NORWAY

## Abstract

**Objectives:**

Research concentrating on continuity of care for chronic, non-communicable disease (NCD) patients in resource-constrained settings is currently limited and focusses on inpatients. Outpatient care requires attention as this is where NCD patients often seek treatment and optimal handover of information is essential. We investigated handover, healthcare communication and barriers to continuity of care for chronic NCD outpatients in India. We also explored potential interventions for improving storage and exchange of healthcare information.

**Methods:**

A mixed-methods design was used across five healthcare facilities in Kerala and Himachal Pradesh states. Questionnaires from 513 outpatients with cardiovascular disease, chronic respiratory disease, or diabetes covered the form and comprehensiveness of information exchange between healthcare professionals (HCPs) and between HCPs and patients. Semi-structured interviews with outpatients and HCPs explored handover, healthcare communication and intervention ideas. Barriers to continuity of care were identified through triangulation of all data sources.

**Results:**

Almost half (46%) of patients self-referred to hospital outpatient clinics (OPCs). Patient-held healthcare information was often poorly recorded on unstructured sheets of paper; 24% of OPC documents contained the following: diagnosis, medication, long-term care and follow-up information. Just 55% of patients recalled receiving verbal follow-up and medication instructions during OPC appointments. Qualitative themes included patient preference for hospital visits, system factors, inconsistent doctor-patient communication and attitudes towards medical documents. Barriers were hospital time constraints, inconsistent referral practices and absences of OPC medical record-keeping, structured patient-held medical documents and clinical handover training. Patients and HCPs were in favour of the introduction of patient-held booklets for storing and transporting medical documents.

**Conclusions:**

Deficiencies in communicative practices are compromising the continuity of chronic NCD outpatient care. Targeted systems-based interventions are urgently required to improve information provision and exchange. Our findings indicate that well-designed patient-held booklets are likely to be an acceptable, affordable and effective part of the solution.

## Introduction

Non-communicable diseases (NCDs) are the leading cause of death and disability across the globe and account for approximately 60% of mortality in India [1]. Due to the rising prevalence of NCDs, low and middle-income countries (LMICs) are having to move from the treatment of communicable disease, typically in single episodes, to focus on long-term management of chronic conditions involving multiple care episodes over long time periods. Such care is more challenging to organise since it must be coordinated across different healthcare professionals (HCPs) often working in different locations.

Chronic NCD management requires effective continuity of care, which is defined as: “the seamless provision of healthcare between settings and over time” [2]. This relies on high-quality information transfer at two levels. At the first level, information must be transferred effectively between HCPs within and between different settings. This is crucial as points of clinical handover, where critical information and responsibility for patient care is transferred from one HCP to another [3], are associated with increased risk to the patient. Such risks are the result of communicative failures and include incorrect treatment, diagnostic delays, life-threatening adverse events and an overall poorer quality and coordination of care [4, 5]. At the second level, efficient information transfer between HCPs and patients is necessary in order to enable patients to become active partners in their NCD management. This healthcare communication is a critical component of patient-centred care, which has been shown to improve clinical outcomes and increase satisfaction with care [6]. This is particularly important during care transitions, as the patient is the sole constant throughout the care process and can provide valuable information to those involved at different stages [7].

The issues regarding transitions in healthcare have been recognised for a long time in high-income countries (HICs). An extensive literature has accumulated over the years describing associated challenges and evaluating interventions to improve them [4–9]. However, a review of the literature has shown that there is currently a dearth of similar research in LMICs generally and India specifically [10]. This is problematic as designing health systems interventions to successfully improve processes such as clinical handover and continuity of care requires elucidation of context-specific challenges [11, 12]. Further, the small amount of LMIC research that has been done has focused on inpatients. Studies concentrating on LMIC outpatient care (particularly in hospitals) are urgently needed as this is where many chronic NCD patients seek regular treatment due to poorly developed primary care systems.

This study was designed to investigate clinical handover and healthcare communication for chronic NCD outpatients with respect to verbal and documented information exchange and medical record-keeping. It forms part of a mixed-methods development project aiming to provide an overview of handover and factors affecting continuity of care for chronic NCD patients in Kerala and Himachal Pradesh states, India [13].

The primary objectives of the study were: 1) To describe clinical handover and healthcare communication practices for chronic NCD patients attending outpatient clinics (OPCs) and 2) To identify barriers to continuity of care for chronic NCD outpatients. A secondary objective was to explore potential interventions to improve the storage and exchange of healthcare information between HCPs and between HCPs and patients.

## Materials and methods

### Overview

We conducted a mixed-methods study comprising structured questionnaires and semi-structured interviews. Qualitative and quantitative data was collected concurrently. Questionnaire data was used to describe the nature and comprehensiveness of clinical handover and healthcare information exchanged between HCPs and between HCPs and patients. Interview data was used to explore experiences and practices of clinical handover and healthcare communication, as well as intervention ideas. The data was then triangulated to elucidate barriers to continuity of care for chronic NCD outpatients.

### Study setting

This study was conducted from December 2014 to November 2015 in seven healthcare facilities: one rural Primary Health Centre (PHC), one rural Community Health Centre (CHC) and one rural secondary-care hospital in Himachal Pradesh state and two rural PHCs, one peri-urban secondary-care and one urban tertiary-care hospital in Kerala state. These settings were selected to represent a range of geographical and public health service settings across India. Government health facilities were the target of this research as this is where many of the most vulnerable patient populations access healthcare and where clinical handover is needed between different levels of healthcare facilities. See S1 Methods for further information regarding the Indian healthcare system and S2 Methods for further information regarding the study settings.

### Ethical considerations

This study was reviewed and approved by the Centre for Chronic Disease Control Independent Ethics Committee, India and the Amrita Institute of Medical Sciences Institutional Ethics Committee, India.

#### Informed consent

Participants who met the inclusion criteria for this study were provided with a study information sheet and were verbally informed by researchers about the purpose of the research. Written consent was obtained from literate patients. For illiterate patients, oral consent was obtained along with a thumb print and signature from a literate witness (e.g. family member or carer) in line with World Health Organisation ethical guidelines [14].

### Patient recruitment

Patients were recruited opportunistically from outpatient clinic (OPC) waiting areas by trained researchers (n = 6). Purposive sampling [15] was used to select adult patients (18yrs+) with one (or more) of the following chronic NCDs requiring follow-up: diabetes mellitus, chronic respiratory disease (CRD), hypertension, or cardiovascular disease (CVD) other than hypertension alone. For both the quantitative and qualitative study components, researchers identified eligible participants by approaching patients/carers and asked them about their health condition/s; patient-held medical documents were also consulted if patients gave their permission for this to be done. Patients were only recruited for the semi-structured interviews if they had not participated in the quantitative questionnaire. This is because participating in the questionnaire could have primed interview participants with pre-prepared and potentially biased responses. This method also ensured that data was gathered from a larger scope of participants.

### HCP recruitment

HCPs were recruited opportunistically to take part in semi-structured interviews from hospitals, primary health centres, or community health centres and were included if they were currently working in an OPC. Due to the busy nature of the health facilities, HCPs who stated that they were too busy to answer questions were deemed as “unavailable” and were not included in the study.

### Qualitative data collection (semi-structured interviews)

Qualitative study participants were recruited until theoretical saturation [16] was reached. Once consent was given, a clinical public health professional (SJ) conducted semi-structured interviews with outpatients in OPC waiting rooms and with HCPs in healthcare facility offices. Qualitative data collection took place in two stages. In the first stage (December 2014 –October 2015), a pre-prepared topic guide was used to guide interviews, which explored clinical handover experiences and practices, healthcare communication (between HCPs and between HCPs and patients) and possible interventions for improving information exchange. Then, on the 11^th^ of October 2015, an expert’s meeting took place in India in order to present some of the preliminary findings and discuss potential interventions.

Representatives from the University of Birmingham, UK and the University of Warwick, UK presented the findings and facilitated group discussions. The participants of the meeting were a range of representatives (n = 27) from the following international, Indian national and state-level organisations: The World Health Organisation (regional and country offices); The World Bank; ACCESS Health International; The Ministry of Health and Family Welfare; The Public Health Foundation of India; The National Centre for Disease Control; The Centre for Chronic Disease Control; The National Health System Resource Centre; The All India Institute of Medical Sciences; Aga Khan Health Services; AMRITA Institute of Medical Sciences and Fortis Hospitals. During discussions there was an overarching consensus that a patient-held booklet for storing and transporting medical documents was likely to be a suitable intervention. This was based on international successes of patient-held maternal and child health documents [17–21] and took into account the delays in developing universal electronic information systems that are accessible across all levels of care. Overall, the booklet was opted as the most pragmatic and affordable intervention to improve information exchange for chronic NCD patients in our study settings. It was also considered to hold potential for improving patient self-management, as booklets could contain disease-specific advice and information.

Therefore, following the meeting the second stage of qualitative data collection (October–November 2015) began. Topic guides were updated to include questions regarding the utility of a patient-held booklet containing detailed healthcare information and researchers were instructed accordingly. In addition, researcher feedback regarding previous interviews indicated that HCPs and patients did not always have a lot of time to be interviewed. Therefore, during the second stage of interviews, if a participant stated that they had limited time to talk then researchers were instructed to interview them using a shortened topic guide; this contained targeted questions on the utility of patient-held medical documents and booklets.

All interviews were conducted and audio-recorded in Hindi, Malayalam, Tamil, English, or a mixture of these languages depending on interviewee preference. Recordings were transcribed verbatim and translated into English as required (SJ).

### Quantitative data collection (structured questionnaires)

Trained social work graduate researchers identified eligible patients and sought written consent for them to take part. Patients were then interviewed by researchers using a questionnaire before and immediately after OPC doctor visits. The first section of the questionnaire (prior to seeing the doctor) covered patient demographics, previous HCP visits and healthcare information provided by previous HCPs. The second section (after seeing the doctor) covered check-up plans and verbal and documented healthcare communication during the OPC visit. Additionally, a checklist was included at the end of each section of the questionnaire to evaluate the contents of patient-held medical documents. See S3 Methods for information regarding the sample size calculation for the quantitative study component.

### Analysis

#### Qualitative data

For qualitative data, Inductive Thematic Content Analysis [22] identified categories and themes; Outpatient and HCP transcripts were analysed separately and themes were then combined. An experienced qualitative researcher (SG) independently assessed the explanatory value of the developing categories against the transcripts.

#### Quantitative data

Descriptive statistics were utilised to outline demographic information and verbal and documented clinical handover and healthcare communication for chronic NCD outpatients.

Whilst it was not possible to assess patient information needs on a case-by-case basis, we aimed to categorise whether patients received all “key” information necessary for facilitating continuity of care both prior to and during OPC visits. Therefore, we selected key contents based on common themes across the literature regarding critical information needed to improve care transitions for patients with chronic/complex care needs [23–25]. This included: diagnosis, medication information (i.e. new prescription and/or details regarding current medication), long-term care advice (i.e. how to manage medication and/or other aspects of management/treatment) and follow-up information (i.e. where to go for the next check-up). For the purposes of this study, this information was considered the minimum required to be provided to each patient to sustain their ongoing management needs.

#### Triangulation

For establishing barriers to continuity of care, convergent triangulation [26] was used to compare and contrast quantitative and qualitative findings.

## Results

### Qualitative data

Table 1 displays participant demographic information. The qualitative study component included 11 outpatients and 22 doctors with various experience and specialties. Analysis revealed 5 major themes (Table 2).

**Table 1:** Qualitative study—Participant demographic information.

Outpatients Characteristic	No. (n = 11)	%
** Sex**		
Female	3	27.3
Male	8	72.7
** Age group**		
18-49yrs	0	0
50-69yrs	6	54.6
≥70yrs	5	45.5
** Language/s spoken in interview**		
English (only)	0	0
Hindi (only)	4	36.4
Malayalam (only)	5	45.5
English & Hindi	1	9.1
Malayalam & Tamil	1	9.1
**Doctors** **Characteristic**	**No. (n = 22)**	**%**
** Sex**		
Female	4	18.2
Male	18	81.8
** Age group**		
18-49yrs	16	72.7
50-69yrs	6	27.3
≥70yrs	0	0
** Job title/position of doctor**		
Medical Superintendent	1	4.6
Consultant	9	40.9
Chief Medical Officer	4	18.2
Medical Officer	3	13.6
General Surgeon	1	4.6
General Medicine	1	4.6
Medical Intern	3	13.6
** Workplace**		
Hospital	17	77.3
CHC	2	9.1
PHC	3	13.6
** Language/s spoken in interview**		
English (only)	20	90.9
English & Hindi (mixture)	2	9.1

**Table 2:** Qualitative themes, categories and illustrative quotes.

Themes	Categories	Illustrative Quotations
**Patient preference for hospital visits**	a) Routine behaviours b) Convenience of hospital location c) Confidence in and familiarity with services and staff	a) OP3: “I come here for all check-ups” b) OP8: “For me, it is convenient to come here as I come to the city for work and I just get check-up also” c) OP1: “…I thought I will show to Dr. A because I believe him”
**System Factors**	d) High patient load at hospital OPCs e) Lack of PHC/CHC medical staff f) Absence of hospital outpatient department medical record-keeping g) Absence of formal clinical handover and referral communication training for doctors h) Absence of structured/standardised referral documents at some facilities i) Basic computerised OPC registration system at some facilities a) Inter-hospital telephone referrals from OPC doctors to jother departments k) Inter-hospital transfer forms at one facility l) PHC NCD register and treatment cards m) PHC referral documents n) Future transition to state-wide paperless/computerised systems	d) Doc15: “OPC… will come around 800–900 and then afternoon is 300” e) OP3: “…why will I go to community health where all doctors are not available” f) Doc4: “Yeah if it’s outpatient we don’t keep record” g) Doc14: “…we have to develop our communication skills ourselves no formal training is there” h) Doc15: “There is no referral format we are only writing in the outpatient ticket” i) Doc 6: “Here outpatients are already computerized…doctor sign in the OPC register and write that OP number…. regarding outpatients that’s all”. j) Doc7: “We call the doctor and discuss the case” k) Doc4: “Yeah there is a inter hospital transfer form” l) Doc17: “… we issue a NCD card… this is the NCD client register… this is the treatment care we give to the patient” m) Doc17: “Yeah we have a referral form… we use a referral form” n) Doc 6: “We have submitted a proposal for paperless computerization system for doctors, so I think state-wide they are planning to do that”
**Inconsistent doctor-patient communication**	o) Inconsistent check-up requests p) Little advice given regarding physical activity, diet and lifestyle q) Sometimes advice is given to patients to bring documents/records	o) OP2: “No they don’t tell us. We come on our own” p) OP3: “I was asked to take less salt, less meat… Doctor didn’t say anything about exercise” q) Doc13: “Some of the time, I fully refuse it… I will not give you any medication unless you bring old record”
**Attitudes towards medical documents**	r) Some patient understanding of the value of keeping medical documents s) Lack of consistent (patient) maintenance and transportation of patient-held medical documents t) Intervention suggestion of a patient-held booklet for holding/transporting medical documents considered acceptable by many patients u) Intervention suggestion of a patient-held booklet for holding/transporting medical documents considered acceptable by several HCPs. v) Doctors’ awareness of the importance of patients keeping/transporting medical documents w) Some doctor preference for paper-based patient-held medical documentation	r) OP 9: “We are afraid sometimes that we might misplace, so we put staple pins and keep all the papers together” s) Doc17: “…patients [that] bring old medical records are few!” t) OP9: “…for me notebook is convenient… it’s much better than holding onto bunch of papers” u) Doc 20: “It is useful. If there are enough funds, it will be useful. Just like discharge we can give some instructions booklet” v) Doc14: “It’s good to have medical records but we don’t get it always” w) Doc 11:” …actually they provided us the computer, then I returned computer. How can I enter? Even I don’t know how to run a computer and all that. If it is paper based it will help”.

#### Theme 1: Patient preference for hospital visits

Overall, most patients preferred using government hospitals for regular check-ups rather than PHCs/CHCs. The reasons they gave were: it is part of their regular routine to go to the hospital, they prefer the central location of the hospital and they have increased confidence in and familiarity with hospital services and staff.

#### Theme 2: System factors

Both patients and doctors described the high patient loads at hospitals OPCs, which resulted in doctors having a short amount of time to see each patient. A contributing factor to this patient rush appeared to be a shortage of healthcare staff at PHCs and CHCs, which resulted in many patients preferring to visit hospital. With regard to referrals, only one doctor mentioned that specific referral documents were available at their PHC facility, while others reported often having to write referral notes on other patient-held documents (e.g. prescription cards) due to an absence of formal documentation. Computerisation of OPC registration systems at some facilities was reported by doctors, but these do not double as a medical record. There was no system of medical record-keeping and retrieval for outpatients at any facility. Additionally, no specific clinical handover or referral training was provided for HCPs at medical school or work. Doctors at two facilities in Kerala reported that there are plans in motion for all government healthcare facilities in the state to transition to “e-health” (i.e. fully computerised health information systems).

Some examples of good handover procedures emerged—such as use of an inter-hospital transfer form at one hospital, some doctors telephoning colleagues in other departments/hospitals to notify them of a referral and one PHC kept an NCD register and each patient was given a treatment card to bring to appointments.

#### Theme 3. Inconsistent doctor-patient communication

Patient reports indicated that the information doctors gave to them was notably inconsistent across OPC appointments. There was a range of ways follow-up check-ups were communicated: some patients were given no instructions and planned to either self-refer to another HCP or return to the same clinic, whereas some were asked to return after a specific amount of time and given medication prescriptions to cover that period. With regard to physical activity, diet and/or lifestyle advice, many patients reported receiving either minimal or none of this type of information. In addition, one patient who recalled receiving a “diet plan” felt that it was not suitable for them because of their socio-economic deprivation.

#### Theme 4. Attitudes towards medical documents

Some patients valued documentation provided by HCPs and kept hold of everything they were given. However, others admitted throwing documents away and some doctors reported that it was uncommon for them to see patients who brought previous healthcare documents. Regarding possible interventions, the majority of patients who were asked expressed positive attitudes towards the introduction of patient-held booklets for storing and exchanging more detailed healthcare information between HCPs and between HCPs and patients.

OPC doctors stressed the importance of patient retention and transportation of medical notes for facilitating continuity of care, but reported that many patients do not bring them. When asked, most doctors supported the intervention suggestion of a patient-held booklet to store and transport documents as they felt it would improve the accessibility and storage of key information. One doctor expressed preference for using paper-based medical documentation rather than computerised documents due to a lack of time and computer skills.

### Quantitative data

#### Demographics

A total of 513 outpatients completed questionnaires. More women (58.1%) participated than men (41.9%). The majority of outpatients were aged 50–69 years (66.1%) and were literate (88.8%) (Table 3).

**Table 3:** Quantitative study–participant demographic information.

	Males (n = 215)	Females (n = 298)	Total (n = 513)
Characteristic	Frequency (%)	Frequency (%)	Frequency (%)
**Age Group (Years)**			
18–49	33 (15.4)	62 (20.8)	95 (18.5)
50–69	138 (64.2)	201 (67.5)	339 (66.1)
≥70	44 (20.5)	35 (11.8)	79 (15.4)
**Level of Education**			
Illiterate	24 (11.2)	57 (19.1)	81 (15.8)
Literate with Partial/Complete Primary School Education	92 (42.8)	125 (42.0)	217 (42.3)
Higher Vocational studies and/or Complete Secondary School Education	80 (37.2)	99 (33.2)	179 (34.9)
Graduate or above	19 (8.8)	17 (5.7)	36 (7.0)
**Employment Status**			
Employed	93 (43.3)	51 (17.1)	144 (28.1)
Unemployed	99 (46.1)	239 (80.2)	338 (65.9)
Retired	23 (10.7)	6 (2.0)	29 (5.7)
Student	0 (0)	2 (0·7)	2 (0.4)
Chronic NCD*			
Chronic Respiratory Disease	64 (29.8)	81 (27.2)	145 (28.3)
Diabetes	78 (36.3)	118 (39.6)	196 (38.2)
Hypertension	80 (37.2)	130 (43.6)	210 (39.0)
Cardiovascular Disease (other than hypertension alone)	81 (37.7)	63 (21.1)	144 (28.1)

*Please note that participants could select more than one answer for this question

#### Sources of referral to the OPC

The most common source of referral to the OPC was patient self-referral (46.2%), followed by referrals from doctors at the same hospital from a previous visit to the inpatient/outpatient department (38.0%) (Table 4).

**Table 4:** Descriptive results–before OPC visits.

Before OPC Visits	No. (n = 513)		%
Source of referral to OPC*	** **		** **
Self-referrals	237		46.2
Government primary-care	50		9.8
Doctor at OPC or inpatient department of this (same) hospital	195		38.0
Other government hospital	38		7.4
Private hospital or nursing home	45		8.8
Private doctor or nurse	4		0.8
Traditional healer / Religious healer	2		0.4
Family or friends	9		1.8
** Brought medical document/s from previous HCP/s to hospital (seen by a researcher)**			** **
Yes	311		60.6
No	202		39.4
** Types of medical documents brought to hospital (seen by a researcher)**	**No. (n = 311)**		**%**
** **Discharge summary	42		13.5
** **OPC document	20	6.4	
** **Prescription card	226	72.7	
** **Formal referral document (i.e. letter/form)	18		5.8
** **Test results	2		0.6
** **Unspecified †	3		1.0
** Contents of document/s from previous HCP/s (checked by a researcher)**	**No. (n = 311)**		**%**
** **Illegible notes	62		19.9
** **Name of doctor/contact at hospital	262		84.2
** **Date	281		90.4
** **Name, age and sex of patient	296		95.2
** **Past medical history for current condition	219		70.4
** **Past medical history for other conditions	71		22.8
** **Patient’s symptoms, signs and problems	181		58.2
** **Tests performed (without results)	60		19.3
** **Tests performed (with results)	187		60.1
** **Diagnosis	283		91.0
** **Medication information	205		65.9
** **Long-term care advice	155		49.8
** **Lifestyle change recommendations (e.g. diet, tobacco, alcohol, exercise, etc.)	116		37.3
** **Follow-up information	163		52.4
** **Unspecified †	13		4.2
** Document/s contained all key information** ¶	**102**		**32.8**
**Did not bring medical document/s from previous HCP/s to hospital (despite having them at home)** §	**No. (n = 513)**		**%**
Yes	201		39.2
No	312		60.8
** Reason for not bringing medical document/s to hospital**	**No. (n = 201)**		
** **Forgot it at home	33		16.4
** **Lost it	12		6.0
** **I've always had it before but the HCPs never used it so I did not bring it this time	45		22.4
** **I didn't think that it was relevant to bring the note/s with me	52		25.9
** **I've never been asked for it here so did not bring it this time	16		8.0
** **My children/spouse handle such documents, so I don't know where they are	14		7.0
** **No data †	29		14.4

*Please note that patients could select more than one answer for this question

† Unspecified/No data = missing responses

¶ Patient-held medical documents containing all of the following: diagnosis, medication, long-term care and follow-up information

§ Patients who reported leaving some/all medical document/s (i.e. anything other than prescription card) from previous HCPs at home

#### Patient-held medical documents brought to the OPC

Over half of all patients (60.6%) brought medical documents to the OPC that they received from previous HCP visits. The most common type of documents brought to OPCs were prescription cards (72.7%). Only 32.8% of patient-held documents contained all four items of key information necessary for facilitating continuity of care (i.e. diagnosis, medication information, long-term care advice and follow-up information). In addition, 201 (39.2%) patients reported that they had left either some or all of their medical documents from previous HCPs at home; in this case “medical documents” were classified as anything other than prescription cards (Table 4).

#### During OPC visits—Nature of OPC documents

The OPC documents given to patients by doctors during outpatient appointments were sheets of paper often provided for other purposes (usually prescription slips or OPC registration papers), on which a HCP had recorded additional notes (e.g. regarding diagnosis, test results, etc.).

#### Information exchange during OPC appointments

Most patients (97.1%) recalled that they had their health condition explained to them during their OPC visit. Only 55.2% of patients recalled receiving both follow-up and medication instructions. All patients received a document with written information during OPC appointments, but only 24.0% of these contained all four items of key information necessary for facilitating continuity of care (i.e. diagnosis, medication, long-term care and follow-up information) (Table 5).

**Table 5:** Descriptive results–during OPC visits.

During OPC Visits	No. (n = 513)	%
Verbal healthcare communication*		
Health condition explained to patient/carer	498	97.1
Patient instructed to return to a HCP for follow-up	435	84.8
Patient given medication instructions (i.e. new prescription and or/continue with previously prescribed medication)	352	68.6
Patient instructed to go for test/s	135	26.3
**Patient received verbal follow-up and medication instructions**†	**283**	**55.2**
** Written information/recommendations provided during OPC visit**		
** **Patient received an OPC document during visit (seen by a researcher)	513	100
** Contents of OPC documents received during visit**	**No. (n = 509)** ¶	**%**
** **Illegible notes	36	7.1
** **Date	491	96.5
** **Name of doctor/contact at hospital	288	56.6
** **Name, age and sex of patient	502	98.6
** **Patient’s symptoms, signs and problems	280	55.0
** **Diagnosis	482	94.7
** **Past medical history for current condition	195	38.3
** **Past medical history for other conditions	56	11.0
** **Tests performed (without results)	46	9.0
** **Tests performed (with results)	224	44.0
** **Medication information	347	68.2
** **Long-term care advice	180	35.4
** **Lifestyle change recommendations (e.g. diet, tobacco, alcohol, exercise, etc.)	225	44.2
** **Follow-up information	256	50.3
** OPC documents contained all key information** §	**122**	**24.0**

*Please note that participants could select more than one answer for this question

†(i.e. “come back for check-up”/”go to local healthcare provider for check-up” and “get some new medication” and/or “continue with old medication”)

¶ Please note that 4 participants did not give permission for the content of their OPC document to be examined

§ OPC documents containing all of the following: diagnosis, medication, long-term care and follow-up information

### Barriers to continuity of care

Table 6 displays barriers to continuity of care that were established following convergent triangulation of the data. Barriers were predominantly systems-based and included: Hospital OPC time constraints, absence of hospital OPC record-keeping, absence of structured patient-held medical documents, absence of clinical handover training and inconsistent referral practices.

**Table 6:** Barriers to continuity of care for chronic NCD outpatients.

Barriers	Data Source (QN / QL*)	Description
Hospital OPC time constraints	Hospital OPC Drs and outpatients (QL)	The large patient loads reported at hospital OPCs meant that doctors did not have much time to see each patient. Doctors reported that this had a negative impact on their ability to provide detailed verbal and documented information when consulting patients. As a result, many patients were not provided with all the key information necessary to facilitate effective continuity of care. A contributing factor to large patient loads appeared to be to patient preference for visiting hospitals due to a lack of resources at local primary health centres.
Absence of hospital OPC record-keeping	Hospital OPC Drs (QL)	No outpatient healthcare records were maintained at the study Hospital OPCs. Therefore, patient medical details could not be accessed at each OPC visit unless patients brought their previous medical documents and/or could recall relevant information.
Absence of structured patient-held medical documents	Hospital OPC Drs and outpatients (QN, QL)	The majority of patient-held medical documents seen by researchers were scraps/sheets of paper with minimal structure. Additionally, some doctors reported not having access to formal referral documents and only one mentioned the use of a specific inter-hospital transfer form. This resulted in inconsistent and often deficient information transfer between HCPs and between HCPs to patients.
Absence of clinical handover training	Hospital OPC Drs and PHC Drs (QL)	Doctors reported that they had not received structured training for clinical handover at medical school or whilst working. Therefore, they had not been provided with the necessary knowledge, skills or structures to effectively and consistently complete clinical handover processes.
Inconsistent referral practices	Hospital OPC Drs, PHC Drs, outpatients (QN, QL)	Doctor reports of varying referral practices indicated an absence of standardised referral systems between primary and secondary government healthcare facilities. Additionally, very few patients arrived at the OPC with formal referral forms and many doctors reported not having access to specific referral documents. This resulted in fragmented information transfer and poor integration between levels of care.

*QN = Quantitative / QL = Qualitative

## Discussion

### Main findings

This study presents mixed-methods data on clinical handover, healthcare communication and continuity of care for chronic NCD outpatients in two states of India. It was found that whilst elements of good clinical handover practice did take place in some primary and secondary-level healthcare facilities, they predominantly happened in isolation and without the existence of structured training or systems to aid their development. It was also found that the patient population attending hospital OPCs seldom received care in the community. These patients were likely to see a different doctor each time they visited the OPC and there were no hospital-based outpatient medical records on which successive HCPs could rely. This meant that communication between HCPs was dependent on patient recall and documented information from previous HCPs that were held and transferred by patients.

These patient-held documents were predominantly re-purposed sheets of paper with minimal structure. However, the contents of these differed substantially between patients and were often insufficient for facilitating continuity of care; only just under a quarter of outpatients received OPC documents containing all the following: diagnosis, medication, long-term care and follow-up information. In addition, a notable proportion of patients did not bring previous documents to the OPC and reports indicated that HCPs did not consistently advise patients to bring them. This meant that HCPs were, at best, having to rely on inadequate and poorly maintained information and, at worst, no information whatsoever.

Notable deficiencies were also evidenced in verbal healthcare communication, with numerous patients reporting either minimal or no provision of lifestyle advice (including diet & activity) during OPC visits. In addition, only just over half of outpatients recalled receiving both follow-up and medication information. Whilst we could not definitively assess the extent to which this was caused by HCP communication or patient recall, the result is equally problematic. This is because many chronic NCD patients left OPC visits unclear about how to effectively manage their condition and engage in self-care activities that could help to prevent further deteriorations. Overall, this shows that continuity of care for NCD outpatients is currently substandard. The finding that key healthcare information is often poorly recorded on patient-held documents is also particularly critical, as there is evidence to indicate that this may compromise patient safety. Research from high-income countries has repeatedly demonstrated a link between deficiencies in documented information transfer between HCPs during care transitions and an increased risk of adverse events, including medical errors and readmissions [27, 28].

### Barriers to continuity of care for chronic NCD outpatients

Barriers to continuity of care found in our study settings were: hospital OPC time constraints, absence of hospital OPC record-keeping, absence of structured patient-held medical documents, absence of clinical handover training and inconsistent referral practices. Whilst our study focused on outpatients and similar LMIC-based studies could not be found, our findings are generally consistent with the limited research from India and other LMICs regarding inpatients. These studies have also found predominantly system-based issues with handover and continuity of care including: poor integration between primary and secondary healthcare facilities, inadequate medical record-keeping, deficient HCP-to-patient communication during care transitions and a scarcity of standardised information exchange systems [29–34]. The descriptions of limited primary care resources are also in line with reports from LMIC literature [35].

The barriers we have found that relate to adverse staff-to-patient ratios at hospital OPCs and limited primary care resources will be challenging to remedy. However, we have also found barriers, such as a lack of record-keeping and an absence of structured patient-held medical documents, which can be remedied at a much lower unit cost. Based on preliminary findings from this study, experts from international, national and state-level healthcare organisations supported the introduction of patient-held record booklets for organising and transporting medical documents; similar patient-held records have proven both affordable and effective for improving continuity of care for maternal and child health globally [17–21, 36, 37]. During subsequent interviews this suggestion was well received by both patients and HCPs. Therefore, this seems to be an acceptable, engaging and relatively inexpensive measure for improving information exchange. These booklets could be specialised to contain structured, disease-specific documentation (e.g. blood pressure charts etc.), which have been proven to improve the quality of recorded healthcare information in both HIC and LMIC settings [38–41] Further, the inclusion of accessible lifestyle advice may help to reduce the burden on government health services by minimising avoidable health crises.

As the utility of booklets would rely on both patient and HCP engagement, it would also be necessary to address the challenges regarding patient understanding, retention, and transportation of medical documents witnessed in this study. Initially, the involvement of both patients and HCPs in the booklet design process would help to create a patient-centred and context-appropriate resource. This is also likely to invoke a sense of ownership amongst its users. The introduction of the booklet could further be accompanied by relevant training and/or education to assist in promoting and normalising utilisation. If necessary, additional incentivisation strategies could be employed to encourage booklet retention such as charging fees for replacement.

Looking further to the future, it should be noted that the implementation of computerised health information systems holds promise for improving the storage and exchange of critical healthcare information; similar systems in HICs and other LMICs have improved guideline adherence, information accessibility and overall efficiency and quality of healthcare [42–44]. Presently in Kerala, electronic information systems are being installed in government primary healthcare facilities and some smaller hospitals [45]. However, this state-wide e-health reform remains in its very early stages and is dependent on strong internet and electrical supplies, which are not available in many areas. This development will also not be able to address the lack of integration between public and private providers that use different information systems, which could further compromise continuity of care for many patients who visit a mixture of providers. Further, patient access to handover and healthcare information may be limited with electronic records. This is because electronic systems require online interfaces for patients to access their information, which also relies on patients owning and using computers/hand-held devices. As far as the authors are aware, this is not currently an area of e-health systems development in Kerala. The authors are also not aware of any plans for electronic health information systems reform in Himachal Pradesh.

Overall, our findings and knowledge of current developments within our study areas suggest that patient-held booklets have great potential to strengthen both current and future health systems. In particular, making patients the custodians of high-quality medical information would facilitate their continuity of care regardless of which HCP they choose to visit. Therefore, further trial and evaluation of this strategy is warranted.

### Strengths and limitations

A key strength of this study is the utilisation of mixed methods, which has provided valuable and in-depth insight to the transfer of critical healthcare information for patients with chronic NCDs. In addition, collecting data from a range of healthcare providers and chronic NCD patients from two diverse states has enhanced the breadth and generalisability of findings. This study is also the first to establish context-specific barriers to aid the targeted improvement of continuity of care for outpatients in an LMIC. However, given the vast size of India and the complexity of the healthcare system, our findings may be difficult to generalise to all areas of India and the fact that private facilities were not assessed is a limitation. In addition, although data saturation was reached and qualitative findings correlated well with quantitative questionnaire data, the absence of participants aged between 18–49 years in interviews may have restricted the representativeness of findings. A lack of adequately recorded inclusion/exclusion rates for participation is also a limitation as this could not be reported.

### Conclusions and next steps

This study is one of the first from an LMIC to systematically report on a range of handover and healthcare communication practices both within and between levels of healthcare. We have found that continuity of care is of poor quality for outpatients with chronic NCDs in our study areas of India. Crucial healthcare information is often not transferred between HCPs and between HCPs and patients, which may be compromising patient safety. The barriers found indicate that these weaknesses are mainly the result of systems-based issues. Ultimately, alongside the development of accessible and fully integrated e-health systems, it would be appropriate to increase the provision of primary and community healthcare resources in line with the declaration of Alma Ata [46]. Clinical handover could then be assisted by technology and formal protocols that strengthen integration [7]. In the meantime, we advocate the production of relatively inexpensive patient-held NCD booklets that will facilitate communication across all levels and types of healthcare.

Finally, given the increasing burden of chronic NCDs in LMICs, we propose that further robust LMIC studies are needed to explore and evaluate the processes involved in clinical handover and continuity of care and identify areas for sustainable intervention.

## Supporting information

S1 Methods Additional information regarding the national healthcare structure in India. (DOCX)

S2 Methods Additional information regarding the study settings. (DOCX)

S3 Methods Sample size calculation for the quantitative study component. (DOCX)
