# Supplementary material for: Investigating clinical handover and healthcare communication for outpatients with chronic disease in India: A mixed-methods study
Source: PLoS One. 2018 Dec 5;13(12):e0207511. doi: 10.1371/journal.pone.0207511 (PMC6281223; doi:10.1371/journal.pone.0207511)
Supplement: S1 Methods — (DOCX) [file pone.0207511.s001.docx]

**S1 Methods. Additional information regarding the national healthcare structure in India**

**National context: Structure of public healthcare system**

The basic structure of the public healthcare system in India is as follows [1]:

- National level: Ministry of Health and Family Welfare.
- State level: State Department of Health and Family welfare in each state.
- Regional level: covers 3 - 5 districts. Headed by State Directorate of Health.
- District level: Middle level management organisation serving as a link between the regional and state structures and the peripheral and PHC structures.
- Sub-divisional/Taluk level: Hospitals/hospitals with specialty care (Taluk headquarters hospitals). Healthcare services are rendered via the office of Assistant District Health and Family Welfare Officer.
- Community level: CHCs that cater for 80,000-120,000 population and PHCs that cover approximately 20,000 – 30,000 population (often upgrades of rural dispensaries).

**Public healthcare across India**

The quality of public healthcare across India varies notably between states and between urban and rural areas. Aside from some pockets of excellence in a select few states, the public sector is generally falling short of meeting the basic healthcare needs of the growing population. Some of the main reasons for this include: services being too far away, a lack of trained personnel and supplies, and limited facility opening times that are often unreliable [2]. With regard to primary health centres, government estimates indicate that 10% are without a doctor, 37% are without a laboratory technician and 25% are without a pharmacist [3]. Issues with public health centres are particularly rife within poor communities, where facilities have been found to be closed more than half the time and lack basic medical supplies. Public facilities are the often the only source of qualified healthcare professionals in rural areas, which is where much of the poor live [2].

**National context: Private healthcare**

Public healthcare in India has lacked funding over a series of decades, resulting from a lack of prioritisation from economic planners. Therefore, the increasing prevalence of chronic, non-communicable diseases alongside unresolved challenges of infectious diseases has placed more strain on public health systems than what can feasibly be managed. The private healthcare sector has subsequently proliferated to meet rising needs, expectations and incomes and surveys indicate that private healthcare providers now dominate service provision. According to reports, public facilities provided just 20% of primary and community-level healthcare services, and 40% of hospital visits in 2004-05 (down from 25% and 60% respectively in 1986-87) [2]. At the higher end of the market the private sector has world class facilities that have grown substantially. As a result, hospital care is now an export sector for medical tourism that cares for approximately 200,000 foreign patients per year [4]. However, private healthcare providers are poorly regulated, with uneven quality across facilities. This is resulting in a large number of private facilities delivering services without appropriate equipment or expertise. Additionally, although visiting private providers is preferential for many, it is common for high out-of –pocket costs to be incurred; more than 40% of all private hospital inpatients have to borrow money or sell assets in order to fund their care [5]. This means that many poorer patients are unable to access healthcare while others fall into poverty as a result of spending.

**References**

1. Care Continuum Alliance, India. India – Health Care System Structure and Organization. 2009. Available from:

http://dmaa.pbworks.com/w/page/17960772/India%20-%20Health%20Care%20System%20Structure%20and%20Organization

1. Joumard I, Kumar A. Improving health outcomes and health care in India. Paris: Organisation for Economic Cooperation and Development (OECD) Working Papers, No. 1184, OECD Publishing. 2015. Available from: <http://www.oecd-ilibrary.org/economics/improving-health-outcomes-and-health-care-in-india_5js7t9ptcr26-en>
2. Ministry of Health and Family Welfare, Government of India. Rural Health Statistics in India. 2012. Available from: https://nrhm.gujarat.gov.in/images/pdf/publications/RHS_2012.pdf
3. Lunt N, Smith R, Exworthy M, Green S, Horsfall D, Mannion R. Medical Tourism: Treatments, Markets and Health System Implications: a Scoping Review. OECD Publishing. 2010. Available from: Paris http://www.oecd.org/els/health-systems/48723982.pdf
4. The World Bank - Health, Nutrition, Population Sector Unit India South Asian Region. Raising the sights: better health systems for India's poor. Available from: <https://openknowledge.worldbank.org/handle/10986/14080>
