# Supplementary material for: Investigating clinical handover and healthcare communication for outpatients with chronic disease in India: A mixed-methods study
Source: PLoS One. 2018 Dec 5;13(12):e0207511. doi: 10.1371/journal.pone.0207511 (PMC6281223; doi:10.1371/journal.pone.0207511)
Supplement: S2 Methods — (DOCX) [file pone.0207511.s002.docx]

**S2 Methods. Additional information regarding the study settings**

**Himachal Pradesh state, India**

Himachal Pradesh is predominantly a rural state in northern India (only 10% of the population lives in urban regions). The average literacy rate is 83.3%, higher than the national average (74%), but rates are notably lower for women compared to men (76.6% vs. 90.8%, respectively) [6]. Private HCPs are less prevalent in Himachal Pradesh compared to other states and public healthcare utilisation remains relatively high [7]. Himachal Pradesh was also recently reported as being the highest spending large state with regard to public health spending per capita [2].

**Kerala state, India**

Kerala is a state in the south-west of India with a greater than national average urban-based population of 47.7%. It has the highest overall literacy rate in India (93.9%; men 96.1%, women 92.1) [8]. There are a relatively large number of government healthcare facilities in Kerala but the healthcare environment is becoming increasingly complex due to a growing popularity and presence of private healthcare providers, predominantly in urban settings [9]. Despite this, public health facilities in Kerala generally play their intended role of being the first point of care and proactively deliver essential services [2].

**References**

1. Census Organization of India. Himachal Pradesh population census data. 2011. Available from: http://www.census2011.co.in/census/state/himachal+pradesh.html
2. Planning Commission, Government of India. Himachal Pradesh Development Report. New Delhi: Academic Foundation; 2005.
3. Census Organization of India. Kerala population census data. 2011. Available from: http://www.census2011.co.in/census/state/kerala.html
4. Planning Commission, Government of India. Kerala Development Report. New Delhi: Academic Foundation; 2008.
