# Supplementary material for: Investigating clinical handover and healthcare communication for outpatients with chronic disease in India: A mixed-methods study
Source: PLoS One. 2018 Dec 5;13(12):e0207511. doi: 10.1371/journal.pone.0207511 (PMC6281223; doi:10.1371/journal.pone.0207511)
Supplement: S3 Methods — (DOCX) [file pone.0207511.s003.docx]

**S3 Methods. Sample size calculation for the quantitative study component**

**Sample size calculation**

We aimed to collect survey data from 385 patients on the basis that this would provide confidence limits of 5% for a variable (such as the proportion of patients receiving complete healthcare information) with a prevalence of 50%, based on the following formula: **((1.96**)**² x** .**5**(.**5))** / (.**05**)**²***.*
